# A Light-Responsive Self-Assembly Formed by a Cationic Azobenzene Derivative and SDS as a Drug Delivery System

**DOI:** 10.1038/srep39202

**Published:** 2017-01-04

**Authors:** Shengyong Geng, Yuzhu Wang, Liping Wang, Tsutomu Kouyama, Toshiaki Gotoh, Satoshi Wada, Jin-Ye Wang

**Affiliations:** 1 School of Biomedical Engineering, Shanghai Jiao Tong University, Shanghai 200240, China; 2 Photonics Control Technology Team, Advanced Photonics Technology Development Group, Center for Advanced Photonics, RIKEN, Wako, Saitama 351-0198, Japan; 3 Shanghai Synchrotron Radiation Facility, Shanghai Institute of Applied Physics, Chinese Academy of Sciences, Shanghai 201204, China; 4 Department of Physics, Graduate School of Science, Nagoya University, Nagoya 464-8602, Japan

## Abstract

The structure of a self-assembly formed from a cationic azobenzene derivative, 4-cholesterocarbonyl-4′-(N,N,N-triethylamine butyloxyl bromide) azobenzene (CAB) and surfactant sodium dodecyl sulfate (SDS) in aqueous solution was studied by cryo-TEM and synchrotron radiation small-angle X-ray scattering (SAXS). Both unilamellar and multilamellar vesicles could be observed. CAB in vesicles were capable to undergo reversible *trans*-to-*cis* isomerization upon UV or visible light irradiation. The structural change upon UV light irradiation could be catched by SAXS, which demonstrated that the interlamellar spacing of the *cis*-multilamellar vesicles increased by 0.2–0.3 nm. Based on this microstructural change, the release of rhodamine B (RhB) and doxorubicin (DOX) could be triggered by UV irradiation. When incubated NIH 3T3 cells and Bel 7402 cells with DOX-loaded CAB/SDS vesicles, UV irradiation induced DOX release decreased the viability of both cell lines significantly compared with the non-irradiated cells. The *in vitro* experiment indicated that CAB/SDS vesicles had high efficiency to deliver loaded molecules into cells. The *in vivo* experiment showed that CAB/SDS vesicles not only have high drug delivery efficiency into rat retinas, but also could maintain high drug concentration for a longer time. CAB/SDS catanionic vesicles may find potential applications as a smart drug delivery system for controlled release by light.

Surfactants, which are usually constituted of a hydrophobic hydrocarbon chain and a hydrophilic head, are classified into non-ionic, anionic, cationic or zwitterionic surfactants. When diluted in aqueous solutions, cationic and anionic surfactant mixtures can form a variety of microstructures, including vesicles^1^,^2^,^3^, rod or worm-like micelles^4^,^5^,^6^,^7^,^8^ and other bilayer lamellar phases^9^,^10^,^11^. Mixtures of single-tailed SDS and single-tailed dodecyl trimethyl ammonium bromide (DTAB)^12^, sodium octyl sulfate (SOS) and cetyl trimethyl ammonium bromide (CTAB)^3^,^13^,^14^,^15^, are two widely investigated catanionic mixtures. The aggregation behavior of cationic/anionic surfactant mixtures is mainly dependent on the ratio of cationic to anionic surfactant, the overall surfactant concentration and the nature of the surfactants, such as the chain length, and the type of polar head^1^,^16^. Among them, catanionic vesicles attracted particular interest because of their vesicular structure, which are similar to liposomes with the ability to encapsulate active molecules^17^. In comparison with liposomes, the preparation procedures of catanionic vesicles are considerably simple and cheap by mixing cationic and anionic surfactants in aqueous solution. And they possess high kinetic stability without degradation or aggregation due to their spontaneous formulation in aqueous solution^18^.

To probe their future pharmaceutical application as delivery vehicles, the interaction mechanisms between catanionic vesicles and cells have been performed on various cell types^19^,^20^. Because the amphiphilic surfactants behaved like bi-tailed lipid molecule^21^, the mechanism between catanionic vesicles and cells includes two pathways, endocytosis and/or fusion^22^,^23^. For example, the SDS-CTAB catanionic vesicles enter the cells via membrane fusion or endocytosis^24^. Azobenzene-containing surfactant/alkyl surfactant catanionic vesicles past the cell membrane through endocytosis^25^. When the catanionic vesicles were incubated with three endocytosis inhibitors to simultaneously inhibit macropinocytosis pathway, clathrin internalization and caveolae uptake, the same inhibition of 50% in vesicle uptake was observed as that incubated at 4 °C, suggesting that macropinocytosis, clathrin and caveolae pathways are the only means of internalisation of endocytosis pathway^20^. In addition, a passive process, membrane fusion occurs within less than 2 min^19^. The encapsulation and interaction of DNA with catanionic vesicles were also widely investigated^26^,^27^,^28^,^29^. The encapsulation and release of model drug from catanionic vesicles were mainly studied in PBS buffer^30^,^31^,^32^,^33^. The *in vitro* drug delivery of catanionic vesicles have also been reported. For example, Texas Red encapsulated inside the aqueous core of catanionic vesicles was delivered into CHO cells^19^. Dew *et al*. incorporated catanionic vesicles in gel formulation, prolonged drug release and decreased *ex vivo* skin penetration rate were achieved^34^. Nevertheless, the *in vivo* drug delivery ability of catanionic vesicles has never been described.

Azobenzenes undergo reversible *trans*-to-*cis* isomerization upon exposure to UV/visible irradiation, and this isomerization can be accomplished in highly viscous solutions, micellar solutions, liquid crystals and even solids^35^. A mixed surfactant system formed from cationic 4,4-bis(trimethylammoniumhexyloxy)azobenzene bromide (BTHA) and SDS has been studied by several groups. Shin and Abbott found its decrease in dynamic surface tensions by UV irradiation^36^. In the study of Bonini *et al*.^37^, small vesicles were the prevalent aggregate when SDS/BTHA was mixed at the molar ratio of 4.8 in lab-adapted conditions. But *trans*-*cis* isomerization reduced the vesicle amount and micelles became the prevailing objects. Similarly, Hubbard and Abbott reported that the vesicles formed by SDS and BTHA were transformed into micellar aggregates after irradiation with UV light^38^. However, all these studies focus on the change in microstructure of aggregates induced by light irradiation. To the best of our knowledge, there is few reports on catanionic surfactant mixtures as drug delivery systems based on light-induced microstructural change.

In our previous work, we have synthesized an azobenzene derivative, 4-cholesterocarbonyl-4′-(N,N,N-triethylamine butyloxyl bromide) azobenzene (CAB), and incorporated it into liposomal membranes to serve as on-off switch of calcein release^39^,^40^. As CAB could be considered as a kind of cationic surfactant, the mixed surfactant system formed from CAB and SDS in aqueous solution will be a novel photo-sensitive catanionic self-assembly system. Mouse fibroblast, NIH 3T3 cells have been widely used to evaluate the *in vitro* cytotoxic effect and cellular uptake of nanoparticles^41^,^42^,^43^,^44^. So NIH 3T3 cells were chosen to assess the cytotoxicity of the blank CAB/SDS system and cellular uptake of rhodamine B (RhB)-loaded system in our present study. In addition, a broad-spectrum anticancer drug, doxorubicin (DOX) was used as the model drug and loaded into the vesicles, so a cancer cell line, human hepatocellular carcinoma cells (Bel 7402) was chosen to evaluate the *in vitro* photo-induced anticancer effect of DOX-loaded system. And, eye is a light-sensitive tissue, it will be a suitable system for investigating the *in vivo* light-responsive property of the CAB/SDS system in the future. Now the challenge of ocular drug therapy is to obtain and maintain a therapeutic level for prolonged period of time^45^. Therefore, rat retina was chosen to study the local drug delivery efficiency of the CAB/SDS system in this study. In our previous study^46^,^47^, the cytotoxicity, intracellular delivery process and drug delivery efficiency of the CAB-liposome were fully studied in Bel 7402 cells, NIH 3T3 cells and rat retinas. Hence, we also use the same cell lines and rat retinas to make a comparison of these two drug delivery systems. Firstly, the structures and photo-induced structural change of the self-assembled surfactant system were investigated. Secondly, the potential application of this system as a novel photo-sensitive drug delivery system were evaluated by loading with a water-soluble fluorescent probe, RhB or DOX.

## Results and Discussion

### Self-assembly of CAB and SDS in aqueous solution

**Figure 1:**
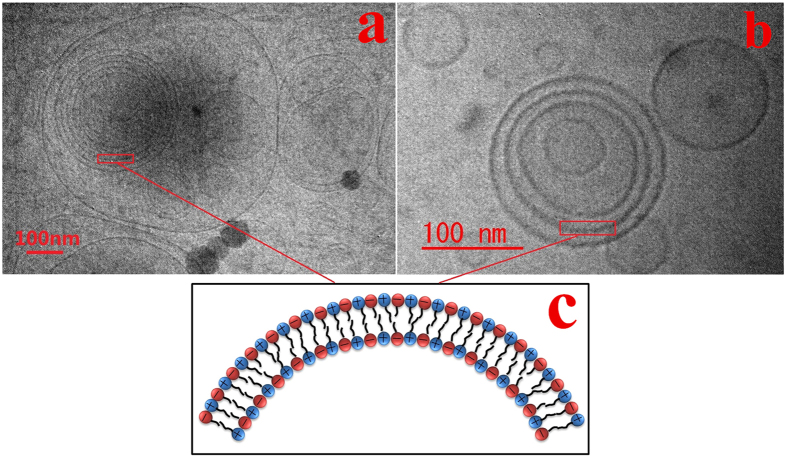
Cryo-TEM images of catanionic surfactants with different compositions of CAB/SDS in solution. (**a**) CAB/SDS = 15/85; (**b**) 30/70; (**c**) schematic illustration of the bilayers of CAB/SDS vesicles.

When pure cationic CAB was dispersed in water at the concentration of 100 mM, a thick liquid like solid was formed. With the addition of anionic SDS, the liquidity of the system was improved and became transparent solution when the relative content of SDS reached 70%. However, for solution having anionic surfactant content of 85%, two phases composed of transparent solution and precipitate were observed. With the aim to develop a nanoscaled drug delivery system, we focused on the transparent solution phase formed by the mixtures of CAB/SDS either at 30/70 (CAB30) or at 15/85 (CAB15) molar ratios. Firstly, dynamic light scattering (DLS) was employed to characterize the size distribution of the self-assembled oppositely charged surfactants. The average diameter was approximately 200–250 nm for both CAB15 and CAB30, as shown in Table 1. The zeta potentials of both CAB15 and CAB30 was negative due to excess amount of SDS. The original morphology of CAB/SDS mixtures was further observed by cryo-TEM, which allows direct visualization of the self-assemblies in solution. As seen in Fig. 1, the catanionic surfactant mixtures were mainly spontaneously organized into spherical vesicles. In addition to unilamellar vesicles, multilamellar structures were also found both in CAB15 (Fig. 1a) and in CAB30 (Fig. 1b). A population of 171 vesicles was analyzed for the CAB15 sample, among which the multilamellar structure occupied 44.4%. For the sample CAB30, 24.6% of the analyzed 195 vesicles were in multilamellar structure. The interlamellar spacing of the multilamellar vesicles was about 10–20 nm. In addition, there was no significant change in the average size of both CAB15 and CAB30 (Fig. S1), indicating that these vesicles were stable for periods as long as 3 months and appeared to be the equilibrium form of aggregation.

Vesicles spontaneously formed in the mixtures of two single-tailed surfactants with opposite charge have been widely observed^1^,^48^,^49^,^50^,^51^,^52^,^53^. It is known that lipids with double hydrocarbon chains such as phospholipids have a tendency to form vesicles, i.e., liposomes. When cationic CAB and anionic SDS were simply mixed in aqueous solution, strong electrostatic interaction between the positively and negatively charged head groups of the two molecules induced formation of ion pairs and a significant decrease in the free energy of a vesicular structure. Since the two tail chains were not bound to the same head group by covalent interaction, this resulting catanionic surfactant could be regarded as a pseudo double-chain surfactant, which behaved like bi-tailed lipid molecule^1^. Then spontaneous formation of closed vesicles occurred via hydrophobic interactions^54^. The bilayers of these CAB/SDS vesicles were illustrated in Fig. 1c.

### Photoisomerization of CAB/SDS vesicle

**Figure 2:**
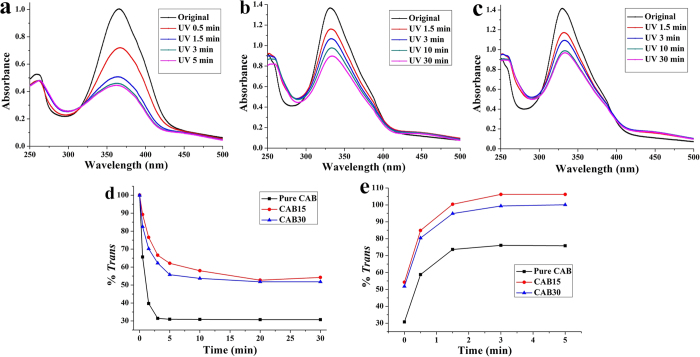
UV-visible absorption spectra of pure CAB (**a**), CAB15 (**b**) and CAB30 (**c**) as a function of UV irradiation time. Percentage of *trans*-sample as a function of irradiation time for *trans*-to-*cis* **(d)** and *cis*-to-*trans* **(e)** isomerization.

Conformational conversion of azobenzene is the fundamental requirement for the self-assemblies containing azobenzene to undergo large structure changes under light irradiation. Figure 2 shows the *trans*-to-*cis* photoisomerization of azobenzene in the CAB/SDS vesicles as verified using UV/Vis absorption. The azobenzene unit of CAB in the absence of SDS was transferred quickly from its *trans* state into *cis* state upon UV light irradiation with the maximum absorption peak at around 365 nm (Fig. 2a). Since the *cis* form of azobenzene has a weak absorption band at 450 nm, irradiation with visible light (λ > 420 nm) caused a relaxation back to *trans* form (Fig. S2a). In the presence of SDS, i.e., CAB15 and CAB30, the same reversible *trans*-to-*cis* isomerization occurred (Fig. 2b and c, Fig. S2b and c). The spectrum reached a photostationary state after 30 min UV irradiation and converted back to the *trans* isomer by visible light illumination for 5 min. It is worth stressing that the absorption peak of azobenzene in CAB15 and CAB30 hypsochromicly shifted to about 332 nm due to the formation of aggregates. Although the *trans* isomer could not be converted completely into the *cis* form under the illumination condition utilized^55^, it seems that existence of SDS inhibited this conversion further. As seen in Fig. 2a–d, the rate and degree of *trans*-to-*cis* isomerization for pure CAB solution were much higher than that for CAB/SDS vesicles. This is because the conformational freedom of CAB molecules was restricted in the bilayer structure formed in CAB/SDS vesicles. In the the case of *cis*-to-*trans* photoconversion, the opposite phenomenon was observed: about 75% *trans* isomer was obtained for pure CAB when the photostationary state was reached (Fig. 2e), owing to the partial overlap between *trans* and *cis* absorption bands^37^. On the contrary, the *cis*-CAB molecules in the CAB/SDS vesicles were rapidly and fully converted to its favored *trans* form in their surrounding environment upon visible light illumination.

### SAXS

**Figure 3:**
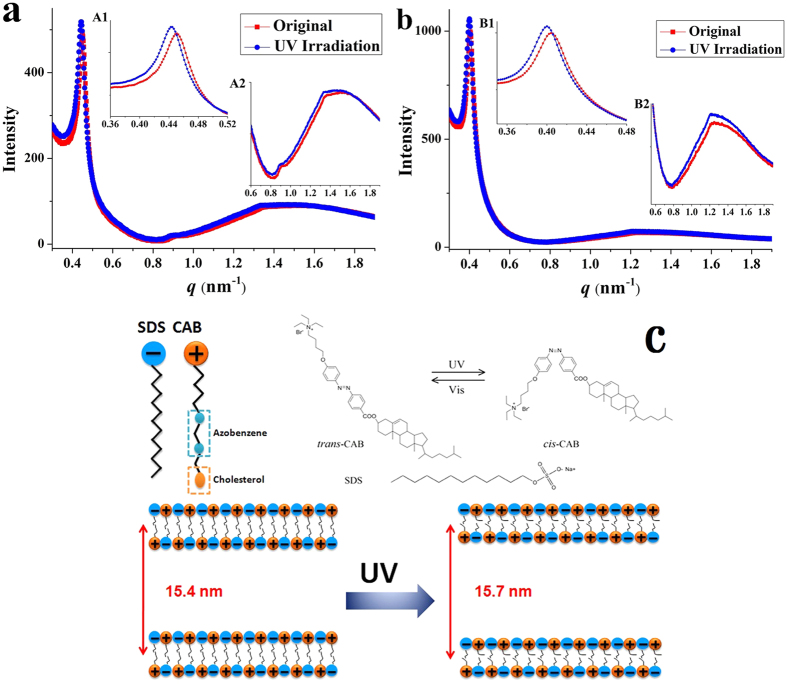
SAXS scattering profiles of mixed CAB15 (**a**) and CAB30 (**b**) aqueous solution before and after UV irradiation. (A1, A2) and (B1, B2), amplification of the scattering peaks of CAB15 and CAB30, respectively. **(c)** Molecular structures of *trans*/*cis*-CAB and SDS, and the schematic illustration of change in interlamellar spacing of CAB30 after UV illumination.

DLS measurement revealed that there was no significant change in the average size between the *trans*- and *cis*-isomers in the two CAB/SDS vesicles (Fig. S3). Much precise understanding of the structural changes in CAB/SDS vesicles upon UV-Vis irradiation needs appropriate techniques, such as synchrotron-based small angle X-ray scattering (SAXS). Figure 3 shows the SAXS profiles of CAB15 and CAB30 before and after UV light irradiation. A sharp scattering peak at *q* = 0.45 nm^−1^ as well as two bumps at 0.91 and 1.36 nm^−1^ were observed in CAB15 sample (Fig. 3a), indicating the presence of an equidistant lamellar structure with the *q* values in the ratios 1:2:3. In the case of CAB30, two peaks at 0.41 and 1.24 nm^−1^ were observed and the middle one was not so clear (Fig. 3b). According to Bragg’s law, *d* = *λ*/(2sin *θ*), the repeat distance (*d*) of bilayers in CAB15 and CAB30 was estimated to be 13.9 nm and 15.4 nm, respectively, which was consistent with the interlamellar spacing of the multilamellar vesicles determined by cryo-TEM. In addition, the result in Fig. S4 showed that the CAB/SDS vesicles were not destroyed or aggregated after they were suffered to synchrotron radiation X-ray irradiation. The reversible *trans*-to-*cis* behavior of azobenzene in CAB15 and CAB30 was not affected either (Fig. S5).

SAXS has been widely used to investigate the internal structures of the spontaneously formed catanionic vesicles. Zhang *et al*. determined the interlamellar spacing of the multilamellar polyhedral vesicles formed by perfluorononanoic acid (C8F17COOH) and tetradecyltrimethylammonium hydroxide (TTAOH) mixtures in aqueous solution by SAXS^56^. Dias *et al*. and Rosa *et al*. determined the structures of DNA-catanionic vesicles complexes based on SAXS measurements^26^,^28^. In the present study, it was observed that the scattering peak shifted to 0.44 nm^−1^ and 0.40 nm^−1^ for CAB15 (Fig. 3A1) and CAB30 (Fig. 3B1), respectively, when exposed to UV irradiation. Correspondingly, the repeat distance was calculated to be 14.1 nm and 15.7 nm. The interlamellar spacing of *cis*-vesicles is expanded by 0.2 nm in CAB15 sample, and 0.3 nm in CAB30 sample.

We used the software “Pymol” to simulate the molecular change of CAB. As shown in Fig. S6, the molecular length of *trans*-CAB was 3.34 nm. It is known that *trans*-to-*cis* isomerization results in shrinkage of azobenzene along the molecular axis from 0.90 nm (*trans*) to 0.55 nm (*cis*)^57^. When *trans*-azobenzene (0.93 nm) was isomerized, its length was reduced to 0.54 nm, resulting in the change of CAB molecular length to 3.04 nm (Fig. S6). That’s to say, the decrease in the molecular length of CAB was 0.3 nm when *trans*-to-*cis* conversion occurred. This result is in consistent with that of the increased interlamellar spacing of *cis*-vesicles calculated from SAXS experiments. So the structure changes of CAB/SDS vesicles observed in SAXS experiments were attributed to the molecular length change of CAB upon UV light irradiation.

Since this thinning is accompanied by an increase in the average surface area of CAB molecule, it would be expected that each bilayer structure in the multilamellar vesicle expands, causing a noticeable increase in the interlamellar spacing. That is to say, when the vesicles were illuminated by UV light, the azobenzene unit of CAB molecule transferred from *trans* to *cis*, resulting in the decrease of bilayer thickness, and a noticeable increase in the interlamellar spacing. To our knowledge this is the first report on *trans*-to-*cis* induced structural change in catanionic vesicles in the resolution of sub-nano level. A schematic illustration described this microstructure change between the *trans*- and *cis*-vesicles was shown in Fig. 3c.

### Photocontrolled release of RhB from CAB/SDS vesicles

Based on the photoresponsive properties and microstructural change investigated above, CAB/SDS vesicles were then considered as a drug delivery system. The CAB/SDS vesicles were closed and contained an inner aqueous compartment, and they showed the ability to entrap water-soluble dyes. Therefore, the drugs can be entrapped in the core of the vesicle if they were hydrophilic. The hydrophilic fluorescent probe rhodamine B (RhB) was used as a model drug, which was loaded into the internal aqueous phase of the vesicles. The encapsulation efficiency (%) of RhB was determined by the following equation: 100 × (*I*_*max*_ − *I*_*o*_)/*I*_*max*_, where *I*_*o*_ is the fluorescence intensity of the CAB/SDS vesicles containing RhB at the initial time, *I*_*max*_ is the fluorescence intensity when all the dye was released after the addition of 0.5% Triton X-100; the encapsulation of RhB was 16.24% ± 1.42 and 29.57% ± 1.45 for CAB15 and CAB30, respectively.

**Figure 4:**
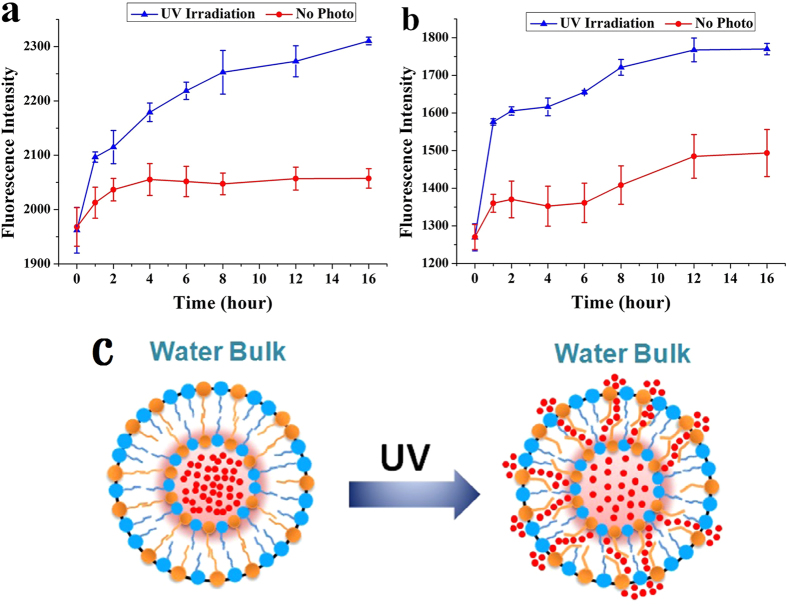
Release profiles of rhodamine B (RhB) from CAB/SDS vesicles (**a**, CAB15; **b**, CAB30) as a function of time after periodic UV light irradiation for 10 min every hour in water, T = 25 °C. Each time point represents the mean ± SD (n = 3). **(c)** Schematic illustration of photocontrolled release of RhB from CAB/SDS vesicles.

Figure 4 shows the release behavior of RhB from CAB15 and CAB30 vesicles by periodical UV irradiation. To prevent the *cis*-CAB converting back to the *trans* form in the dark, the samples were treated with UV light 10 min every hour. The amount of RhB released out from the vesicles into the external medium was proportional to the change in fluorescence intensity. Apparently, the RhB release rate greatly increased after UV light irradiation both in CAB15 (Fig. 4a) and CAB30 (Fig. 4b). A burst release occurred upon UV irradiation at the first hour and then slower release was kept sustained up to 16 h. Compared to CAB15, more dye was released at the first hour in CAB30. The higher percentage of multilamellar structure in CAB15 (~44.4%) might decrease the leak of RhB to the external solution to some extent. So, the releasing profile of RhB is much ideal than that of CAB30. Spontaneous release of RhB could be observed in the group without UV irradiation. We found that the unencapsulated RhB could not be completely removed through dialysis. Thus, RhB absorbed on the surface of the vesicles diffused to the outside medium when the samples were dispersed in aqueous solution. That’s to say, the leakage of RhB in the group without UV irradiation was not entirely attributed to the burst release. It is less possible that the *cis*-isomer of azobenzene moiety in CAB can induce spontaneous release of RhB, as *trans*-isomer is the thermo-dynamically stable form of azobenzene derivatives. Overall, the bent *cis* isomer of CAB molecules created looser space than the straight *trans* structure, leading to diffusion of RhB through the micropores under well controlled UV irradiation (Fig. 4c).

The closed structures spontaneously formed in cationic-anionic surfactant mixtures represent a new way of encapsulation and controlled release. Hargreaves and Deamer reported the first encapsulation experiments of CTAB/SDS system in 1978^58^. They found that temperature was a predominant factor concerning the entrapment/release ability. The catanionic vesicles obtained were impermeable to glucose when heated up to 47 °C, whereas degenerated into membrane fragments once cooling. Cetyltrimethylammonium tosylate and sodium dodecylbenzenesulfonate (CTAT/SDBS) mixtures were also investigated to encapsulate glucose, but the release experiments were not carried out^50^,^59^. In the investigation of Kondo *et al*.^60^, didodecyltrimethylammonium bromide (DDAB)/SDS vesicles were prepared in glucose aqueous solutions and the unencapsulated glucose was removed through dialysis. Although the release of glucose could be induced by addition of Triton X-100, the release rate can’t be adjusted. To the best of our knowledge, this study is the first report of catanionic surfactants based vesicles to realize sustained photocontrolled release of loaded molecules. That may find potential applications as smart carriers for the controlled drug delivery system.

### Evaluation of the cytotoxicity of CAB/SDS vesicles

**Figure 5:**
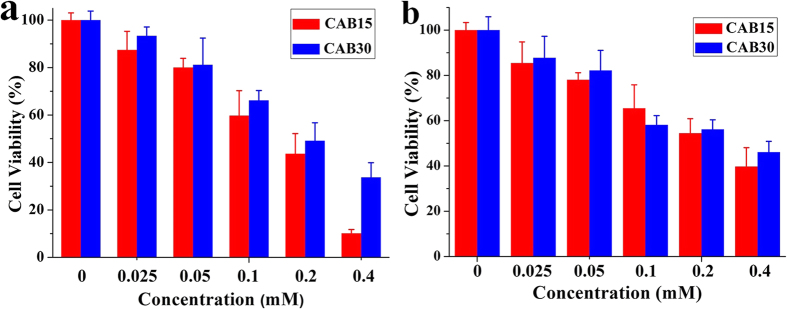
Cytotoxicity of the CAB/SDS vesicles at 24 h treatment with NIH 3T3 cells (**a**) and Bel 7402 cells (**b**). Cell viability was assessed by the MTT assay (n = 6).

Safety is the necessary point when investigating the potential use of the CAB/SDS catanionic vesicles as a drug delivery system. The cytotoxicity of CAB15 and CAB30 vesicles was evaluated against either NIH 3T3 cells or human hepatic carcinoma cell line Bel 7402, as shown in Fig. 5. For both CAB15 and CAB30, there was no significant cytotoxicity at or below the concentration of 50 μM, and a pronounced cytotoxicity at a concentration as high as 400 μM was observed. It should be noted that more than 90% cells survived even if the concentration of CAB was increased to 480 μM (Fig. S7b), while SDS accounted for about 70% mortality rate of the cells at 400 μM (Fig. S7a). Therefore, the cytotoxicity of the CAB/SDS vesicles especially at high concentration was mainly due to the toxicity of SDS. Russo *et al*. analyzed the cytotoxic action of SDS/CTAB and SDS/DDAB vesicles on HEK-293 cells^23^. The survival rate of the cells was not more than 60% after 4 h exposure to SDS/CTAB vesicles, while that was only 20% for SDS/DDAB vesicles treated cells. They ascribed this high toxicity to the CTAB or DDAB component: individual surfactant CTAB at the concentration of 25 μM induced more than 60% mortality rate; around 80% cells survived even when SDS was up to 100 μM. The individual CAB exhibited no significant cytotoxicity even if the concentration was increased to 480 μM (Fig. S7). Therefore, our CAB/SDS vesicles were much less toxic than the SDS/CTAB and SDS/DDAB vesicles. Aiello *et al*. also reported that SDS/CTAB vesicles showed pronounced cytotoxicity eve at a concentration as low as 25 μM^61^.

### *In vitro* photoinduced cytotoxicity assay

**Figure 6:**
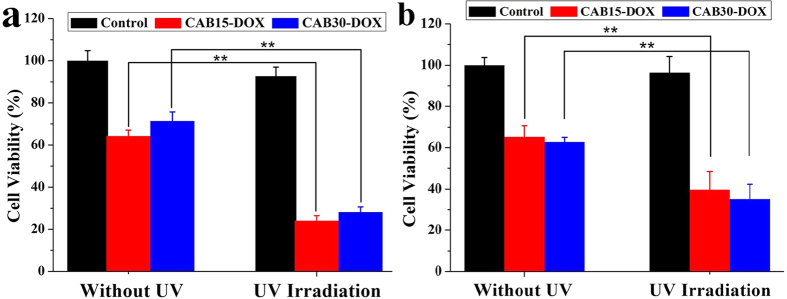
Cytotoxicity of DOX-loaded CAB/SDS vesicles against NIH 3T3 cells (**a**) and Bel 7402 cells (**b**) after 24 h incubation with or without UV irradiation (10 min every 4 hour, total 6 cycles). The concentration of DOX was 20 μM and the cell viability was determined by MTT assay. Data are shown as mean ± SD (n = 6). **p < 0.01 (Student’s t test).

Photo-triggered intracellular drug release from the CAB/SDS vesicles could be evaluated with the combination of cytotoxicity of DOX-loaded vesicles with and without UV irradiation. Figure 6 showed that the cytotoxicity of DOX loaded-CAB/SDS vesicles was markedly enhanced (*p* < 0.01) following UV irradiation in both NIH 3T3 cells and Bel 7402 cells. Cell viabilities were about 60% after 24 h incubation with CAB15-DOX or CAB30-DOX without UV irradiation. In comparison, they decreased by more than 40% with UV irradiation (UV irradiation itself caused about 8% cell death, Fig. 6a and b). UV light induced a higher cytotoxicity of NIH 3T3 cells than Bel 7402 cells, indicating the different sensitivities against DOX. The results presented in this study indicated that the spontaneously formed CAB/SDS vesicles could serve as a promising light-responsive drug carrier.

### Cell uptake of CAB/SDS vesicles loaded with RhB

**Figure 7:**
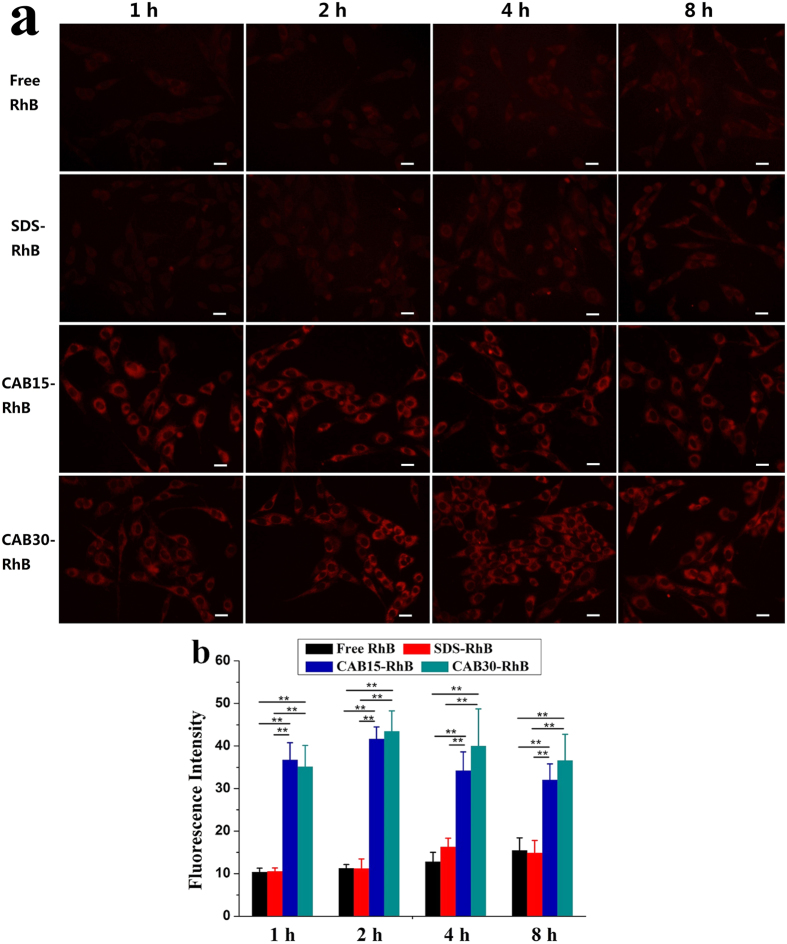
Uptakes of free RhB, SDS-RhB and RhB loaded CAB/SDS vesicles by NIH 3T3 cells. **(a)** Fluorescence images taken by Olympus IX71 at different incubation times. Scale bars are 20 μm. **(b)** Mean fluorescence intensity of the treated cells at different time (n = 6). CAB15 and CAB30 show significant differences compared to free RhB and SDS-RhB, respectively (^**^p < 0.01, Student’s t test).

Figure 7 shows the interaction of NIH 3T3 cells with CAB/SDS catanionic vesicles labelled with a fluorescence probe RhB. Compared to free RhB and SDS-RhB mixtures, the CAB/SDS vesicles (both CAB15 and CAB30) treated cell displayed much higher fluorescence intensity (*p* < 0.01). The cellular uptake process of RhB in different samples was further analyzed through quantitative fluorescence intensity, as shown in Fig. 7b. Both free RhB and SDS-RhB diffused into cell cytoplasm at 1 h and gradually accumulated during 8 h incubation, but the amount of RhB uptaken by cells was quite low. However, a stronger and quicker interaction with the cells could be observed in the case of CAB/SDS catanionic vesicles. The uptake of RhB loaded in CAB15 and CAB30 reached the maximum after 2 h incubation and maintained high levels until 8 h.

Catanionic vesicles have been reported to interact with various types of cells through fusion and/or endocytosis^19^,^20^. Mauroy *et al*. simultaneously labeled the amphiphilic bilayers and the inside aqueous core with different fluorescence probes to study the cellular uptake process of lactose-derived catanionic vesicles^19^. They found that spontaneous membrane fusion occurred within less than 2 min at 37 °C, followed by endcytosis after 15 min of incubation. But the vesicles in their research were positively charged, while our CAB/SDS vesicles were negative. The surface charge of drug delivery systems is an important factor affecting the interaction between cells. Low incubation temperature (4 °C) has been used to block active processes corresponding to endocytosis process^62^, which was strongly temperature dependent. But fusion is not inhibited at low temperature since it is a passive process^62^. Figures S8 and S9 showed the vesicle/cell interactions observed by confocal microscopy. Compared to the cells incubated at 37 °C, the fluorescence of both CAB15-RhB and CAB30-RhB treated cells was much weaker at 4 °C even after 60 min. Therefore, active endocytosis was shown to play a key role in the CAB/SDS vesicles’ entry into cells. To be a nanoscaled drug carrier, CAB/SDS system could greatly improve the delivery efficiency of the entrapped molecules into cells.

### RhB delivery into rat retina

**Figure 8:**
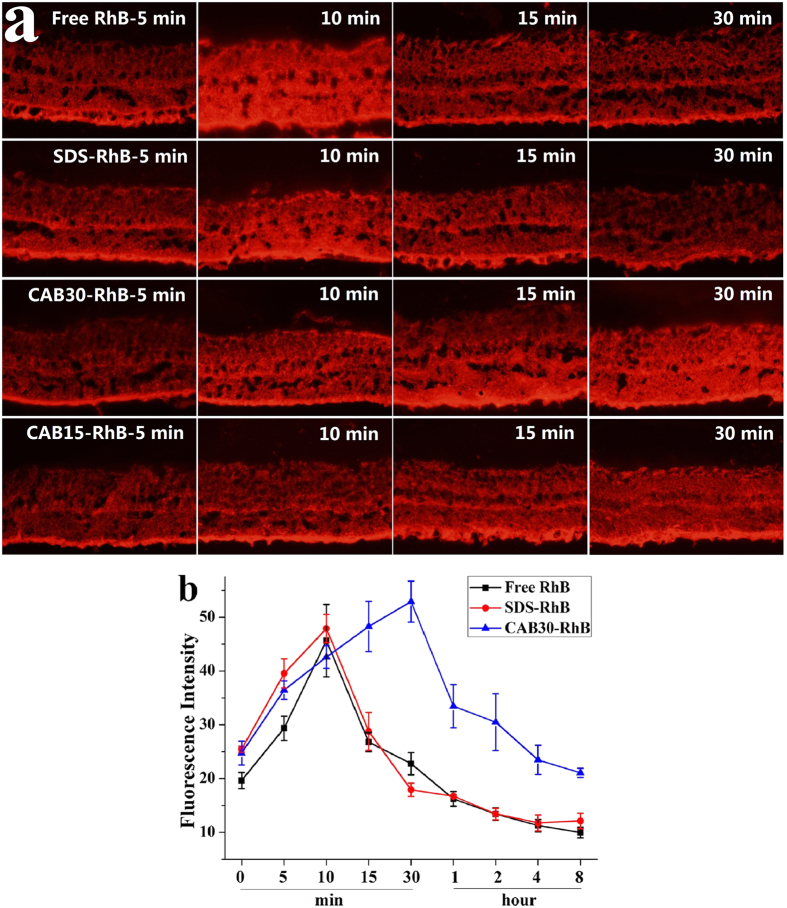
Delivery of RhB by the CAB/SDS vesicles into rat retina. **(a)** Fluorescence images of retinal cryosection treated with free RhB, SDS-RhB, CAB30-RhB and CAB15-RhB after different times. **(b)** Fluorescence intensity of the retinas calculated by Image Pro Plus during 8 h treatment with free RhB, SDS-RhB and CAB30-RhB (n = 3).

CAB/SDS catanionic vesicles loaded with RhB were further administrated into rat retina by intravitreous injection to investigate the *in vivo* drug delivery behavior. Figure 8a showed the fluorescent staining of the inner and outer segments as well as the pigmented epithelium layer of SD rat retinas after administration. In free RhB and SDS-RhB treated eyes, the fluorescence intensity reached the maximum after 10 min of administration and decreased quickly from then on any fluorescence was hardly observed one hour later. That’s to say, once free RhB was injected into the eyes, it diffused into retinas within 10 min and then was removed rapidly through circulation. In addition, SDS had no influence on the delivery of RhB into retina. In the case of CAB30-RhB treated eyes, on the other hand, the fluorescence intensity reached to the maximum 30 min later and then kept much high level of RhB in comparison to the free RhB and SDS-RhB treated eyes up to 8 h (Fig. 8b). CAB15-RhB also showed the same tendency as CAB30-RhB (Fig. 8a). The cholesterol group in CAB may play a key role in the sustained release function. As a conclusion, the CAB/SDS vesicles not only have the high drug delivery efficiency into rat retina but also could maintain high drug concentration for a longer time duration. They may be a promising drug delivery system that could be employed to improve the distribution in ocular drug delivery to the retina.

In our previous study, we investigated the delivery ability of DOX by CAB-liposomes into rat retina^46^. DOX was mainly distributed in the ganglion cell layer and the inner nuclear layer of retina; and the fluorescence intensity increased to the maximum firstly and then decrease gradually. But, in the case of RhB, it distributed throughout the retina after 15 minutes, and then was cleared rapidly. Similar results were reported by Kaiser *et al*.^63^; the rodaminelabeled liposomes-treated retinas showed the most intense fluorescence after 15 minutes, but little fluorescence was observed after 1 hour of intravitreal injection, reflecting rapid clearance of rodamine.

As we know, the poor tissue penetration and potential damage of UV light may limit azobenzene related applications in biomedical field. However, with the development of optical techniques, investigators can now deliver light to any area of the brain, whether the surface or the deep tissues, in freely moving mammals^64^. In addition, recent advances in light source such as two-photon excitation and NIR-to-UV up conversion may address the bottleneck toward demonstrating the clinical feasibility^65^,^66^,^67^,^68^. Actually we are now developing a two-photon laser setup with our cooperator to solve the problem when using in clinic. One of the possible application of our system may be used as the ocular drug delivery system for eye diseases. We conducted *in vivo* experiments in present study and the results revealed that the CAB/SDS vesicles had high drug delivery efficiency into rat retina. Our next goal is to investigate the light-controlled drug release in retina. Another possible application is in the treatment of certain skin diseases, such as psoriasis and vitiligo, and wound care. Because ultraviolet phototherapy has been shown to be extremely effective for these diseases^69^,^70^,^71^. If the drugs for the above skin diseases were loaded into the vesicles, UV irradiation and the induced drug release may play dual therapeutic effects.

## Conclusions

In summary, we synthesized a cationic azobenzene derivative, CAB. When it was mixed with anionic SDS in aqueous solution, they self-assembled into unilamellar and multilamellar vesicles. Owing to the photochemical properties of azobenzene, the vesicles isomerized from *trans* to *cis* upon UV irradiation and converted to the *trans* form under visible light illumination. This feature made these aggregates a good candidate for a photosensitive drug carrier. To further explore the influence of such photoconversion on the microstructure change of these catanionic aggregates, we performed SAXS and calculated the 0.2–0.3 nm increase in the interlamellar spacing of the multilamellar from the scattering peak shift. Subsequently, the vesicles loaded with RhB were prepared to assess the photocontrolled release from these vesicles imposed by *trans*-to-*cis* isomerization. The release behavior of RhB could be well controlled by UV irradiation from both CAB15 and CAB30. To the best of our knowledge, this was the first report of light-triggered release from such spontaneously formed vesicles. *In vitro* toxicity studies demonstrated that the DOX-loaded CAB/SDS vesicles under UV irradiation exhibited higher cytotoxicity than that of the nonirradiated ones. Furthermore, the vesicles had a strong and quick interaction with NIH 3T3 cells and delivered much more RhB molecules into the cells. *In vivo* experiments revealed that the CAB/SDS vesicles have high drug delivery efficiency into rat retina. Overall, our study showed that these catanionic vesicles have potential application as a smart photosensitive drug delivery system.

## Materials and Methods

### Materials

Sodium dodecyl sulfate (SDS) was purchased from Amresco (Solon, OH, USA). RhB was purchased from Tiantai Fine Chemicals CO., LTD (Tianjin, China). Doxorubicin (DOX) was purchased from Melone Pharmaceutical Co., Ltd (Dalian, China). The azobenzene derivative, CAB was synthesized as described before by our group^39^.

### Preparation of CAB/SDS system

Stock solutions of SDS and CAB were prepared by weighing appropriate quality of these two compounds and dissolving in Milli-Q water. To prepare catanionic self-assemblies, CAB and SDS stock solutions were mixed at the molar ratio of CAB/SDS = 15/85 or 30/70, which are hereafter referred as CAB15 or CAB30, respectively. The total concentration of the two surfactants was 100 mM. Then the mixture samples were stirred by vortex mixer for 1 min. The samples were left to equilibrate one week in the dark at room temperature before any experiment was performed on the solutions. To load the fluorescent dye (RhB or DOX), Milli-Q water was replaced with RhB or DOX solution (3 mM, in Milli-Q water). After the vigorous mixing with a vortex mixture and followed by equilibration in the dark, the solution was dialyzed against Milli-Q water to remove unloaded dye.

### Characterization

The dynamic laser scattering (DLS) measurement was carried out on a Malvern Zetasizer 3000 HAS (Malvern Instruments Ltd, UK) at a scattering angle of 90°. The wavelength was set to 633 nm and the temperature was controlled to 25 °C. Size distribution was evaluated with polydispersing index (PdI).

Cryogenic transmission electron microscopy (cryo-TEM) investigations were performed with a JEM2010 instrument (Jeol, Tokyo, Japan) operating at 200 kV. A suspension of the samples was dropped on a carbon-coated grid; the excess liquid was removed by filter paper. Then the grid was flashed-cooled with liquid propane at 83 K and transferred to a cold stage at 100 K in a transmission electron microscope for examination.

### Synchrotron radiation small angle X-ray scattering (SAXS)

SAXS measurements were performed on the beamline (BL16B1) at Shanghai Synchrotron Radiation Facility at 25 °C. The wavelength of the X-ray (λ) was 0.124 nm and the data accumulation time of each sample was 300 s. The two-dimensional (2D) SAXS patterns were recorded by a Mar165 CCD, the resolution of which was 2048 × 2048 pixels with pixel size 80 μm × 80 μm. The distance from sample to detector was 1920 mm (calibrated by beef tendon standard). The SAXS pattern was analyzed by Fit2D software (European Synchrotron Radiation Facility) in terms of the scattering vector *q* = 4π sin *θ*/*λ*, where 2θ is the scattering angle and λ is the X-ray wavelength.

### Photoisomerization of the samples

The sample was diluted to an appropriate concentration in a quartz cuvette and irradiated by a 400-W high-pressure Hg lamp (LCE-9, Zhengzhou, China). A bandpass filter (λ_T_ = 275–400 nm) was used for UV light, and a cut-off filter (λ > 420 nm) (Fujifilm, Japan) was used for visible (Vis) light. The sample-to-light source distance was 15 cm. The UV-Vis spectra of the samples after various periods of illumination were recorded by a UV-Vis spectrophotometer (U3010, HITACHI, Japan). The isomerization rates were calculated according to the following formula: % *trans* = 100 × A_t_/A_o_, where A_o_ is the absorbance at the maximum absorption wavelength (λ_max_) in the initial state and A_t_ is the absorbance at λ_max_ at an accumulated exposure time (t) to UV light.

### Photocontrolled release of RhB from the CAB/SDS system

The CAB/SDS self-assembly, prepared by using 3 mM RhB solution, were put into a dialysis tube (MWCO 8000–14000 Da, Greenbird, Shanghai, China) for dialysis, followed by being transferred into 500 mL of water solution to remove the unloaded RhB. The samples were transferred into a quartz cuvette and exposed to UV light irradiation 10 min every hour, then they were kept in dark at room temperature (25 °C). The samples that were kept in dark without any UV illumination served as the control group. The release behavior of RhB from CAB/SDS sulution was monitored by measuring the fluorescence intensity of the sample using a fluorescence spectrophotometer (F-2500, Hitachi, Japan) at 1, 2, 4, 6, 8, 12 and 16 h.

### Evaluation of cytotoxicity

NIH 3T3 cells and Bel 7402 cells were cultured in DMEM or RPMI 1640 medium, respectively, supplemented with 10% heat-inactivated new born calf serum under humidified conditions at 37 °C and 5% CO_2_. The cells were seeded in a 96-well plate at a density of 5 × 10^4^ cells/mL and cultured for 24 h. Then, the culture medium was replaced with such medium containing a series of concentration of CAB/SDS system (CAB15 or CAB30). After another 24 h incubation, this medium was removed and a medium containing 10% 3-(4,5-dimethylthiazol-2-yl)-2,5-diphenyl tetrazolium bromide (MTT) stock solution (5 mg/mL) was added to each well, followed by incubation for 4 h. After removing the medium, 150 μL of dimethyl sulfoxide (DMSO) was added to each well to dissolve the blue formazan crystals converted from MTT. The relative cell viability (%) was determined by comparing the absorbance at 490 nm. Data are presented as average ± SD (n = 6).

### *In vitro* photoinduced cytotoxicity assay

The CAB/SDS self-assemblies loaded with DOX were prepared in 3 mM DOX solution to study the light-triggered drug release in cells. NIH 3T3 cells and Bel 7402 cells were seeded and cultured the same as described above. After 24 h incubation, the culture medium was replaced with such medium containing CAB15-DOX or CAB30-DOX with DOX concentration of 20 μM. The cells were irradiated by UV light for 10 min every 4 h. Meanwhile, the cells treated with the same samples but without UV irradiation were set as the control. MTT assay was carried out 24 h later. Data are presented as average ± SD (n = 6).

### Uptake of CAB/SDS self-assemblies by cells

NIH 3T3 cells were seeded in a 96-well plate (5 × 10^4^ cells/mL) and cultured for 24 h. To perform cell interactions studies, the culture medium was then replaced with a medium containing free RhB, SDS-RhB, CAB15-RhB or CAB30-RhB. The concentration of RhB was consistent as characterized by the fluorescence intensity. After incubation for 1, 2, 4 and 8 h, the cells were gently washed by PBS for three times and then observed by fluorescence microscope (Olympus IX71, Olympus, Japan). The fluorescence intensity was analyzed by Image Pro Plus (Media Cybernetics, USA).

### Animals

All animal studies were approved by the Institutional Animal Care and Use Committee of Shanghai Jiao Tong University (Shanghai, China). All of the experimental procedures were performed in accordance with the protocols and ethical regulations approved by the Institutional Animal Care and Use Committee of Shanghai Jiao Tong University. Sprague Dawley (SD) rats (female, ~200 g) were obtained from SLAC Laboratory Animal Co., LTD (Shanghai, China).

### RhB delivery into rat retinas

The animal experiment was divided into short-term delivery (0 to 15 min) and long-term delivery (15 min to 8 hours). The procedures of intravitreal injection and retina separation have been described in our previous study^46^,^47^. Briefly, 10 μL of free RhB was injected into the left eyes, the right eyes were injected with SDS-RhB or CAB30-RhB (10 μL). The concentration of RhB was adjusted to 25 μM for all the samples before vitreous injection. For short-term delivery, the time points were 5 min, 10 min and 15 min after injection, while for long-term delivery, they were 15 min, 30 min, 1 h, 2 h, 4 h and 8 h after injection. Then eyeballs of the rats were taken off and fixed in 4% polyformaldehyde for 1 h. Twenty micrometer cryosections of the treated retinas were collected on gelatin-coated slides by Leica CM3050 S. Then, the sections were observed by fluorescence microscope (Olympus BX51, Olympus, Japan) and the images taken were used for fluorescence intensity analysis by Image Pro Plus (Media Cybernetics, USA). The exposure time and other parameters during the process of taking photos were kept consistent. Data are presented as average ± SD (n = 3).

## Additional Information

**How to cite this article:** Geng, S. *et al*. A Light-Responsive Self-Assembly Formed by a Cationic Azobenzene Derivative and SDS as a Drug Delivery System. *Sci. Rep.* **7**, 39202; doi: 10.1038/srep39202 (2017).

**Publisher's note:** Springer Nature remains neutral with regard to jurisdictional claims in published maps and institutional affiliations.

## Supplementary Material

Supplementary Information

## Figures and Tables

**Table 1:** CAB/SDS composition and characterization (n = 3).

Sample	Composition (molar ratio)	Size (nm)	PdI	Zeta Potential (mV)
CAB15	CAB/SDS = 15/85	265.0 ± 7.6	0.26 ± 0.02	−86.8 ± 3.6
CAB30	CAB/SDS = 30/70	234.0 ± 5.0	0.22 ± 0.04	−72.8 ± 2.8
