# Supplementary material for: A Light-Responsive Self-Assembly Formed by a Cationic Azobenzene Derivative and SDS as a Drug Delivery System
Source: Sci Rep. 2017 Jan 4;7:39202. doi: 10.1038/srep39202 (PMC5209711; doi:10.1038/srep39202)
Supplement: Supplementary Information [file srep39202-s1.pdf]

Supporting Information

A Light-Responsive Self-Assembly Formed by a Cationic Azobenzene Derivative and SDS as a Drug Delivery System

Shengyong Geng[1, 4], Yuzhu Wang[2], Liping Wang[1], Tsutomu Kouyama[3], Toshiaki Gotoh[3], Satoshi Wada[4], and Jin-Ye Wang[1, *]

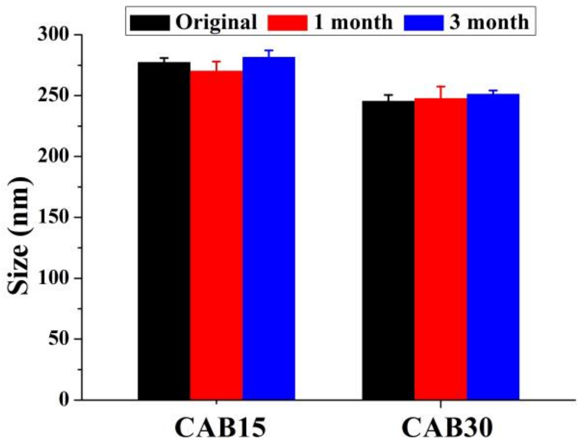

Figure S1. The size change of CAB/SDS vesicles. The samples were kept in distilled water in dark at room temperature (25 °C), and 1 mL of each sample was picked out to measure its size after 1 month and 3 months (n = 3).

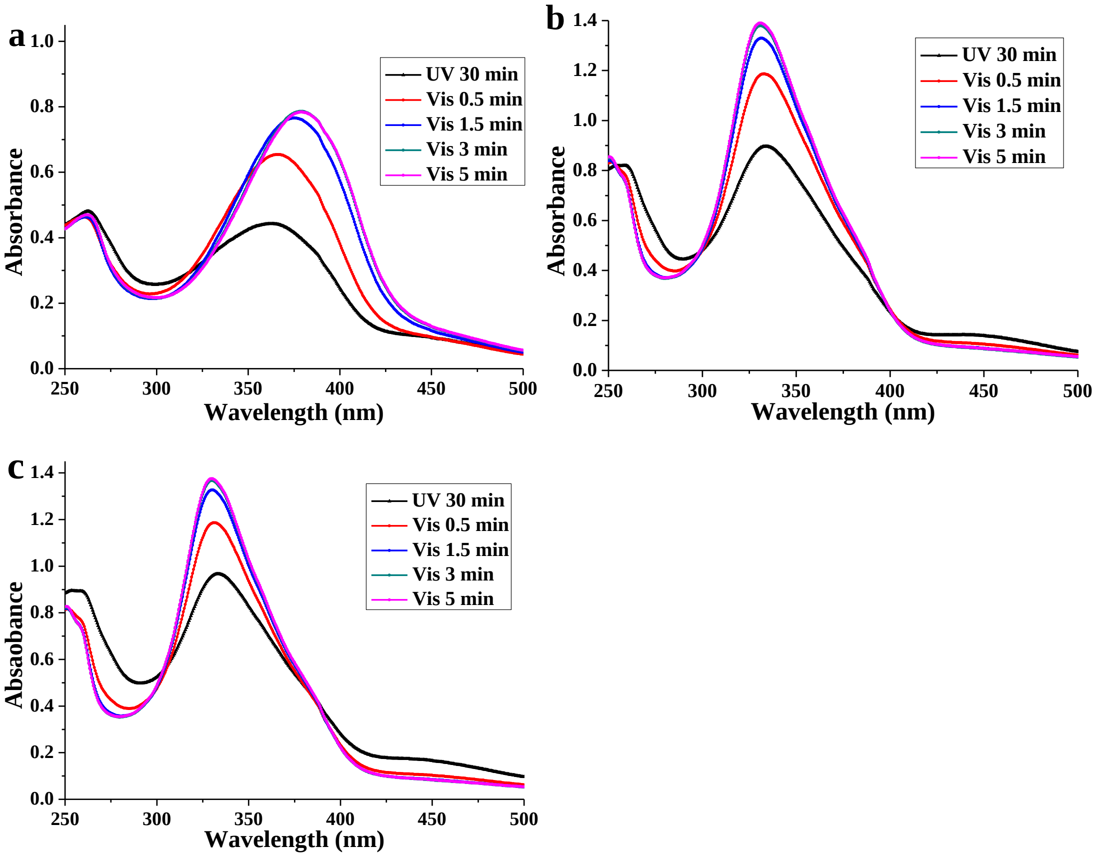

Figure S2. UV-visible absorption spectra of pure CAB (a), CAB15 (b) and CAB30 (c) as a function of visible light irradiation time.

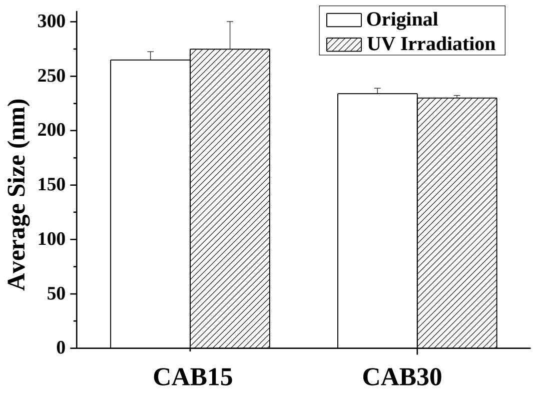

Figure S3. The size change of CAB/SDS vesicles before and after UV light irradiation (n = 3).

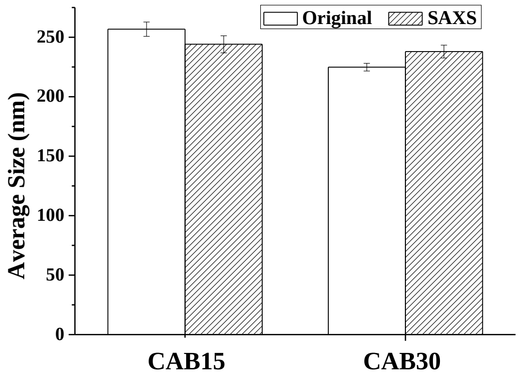

Figure S4. The size change of CAB/SDS vesicles before and after synchrotron radiation small-angle X-ray scattering (SAXS) measurements (n = 3).

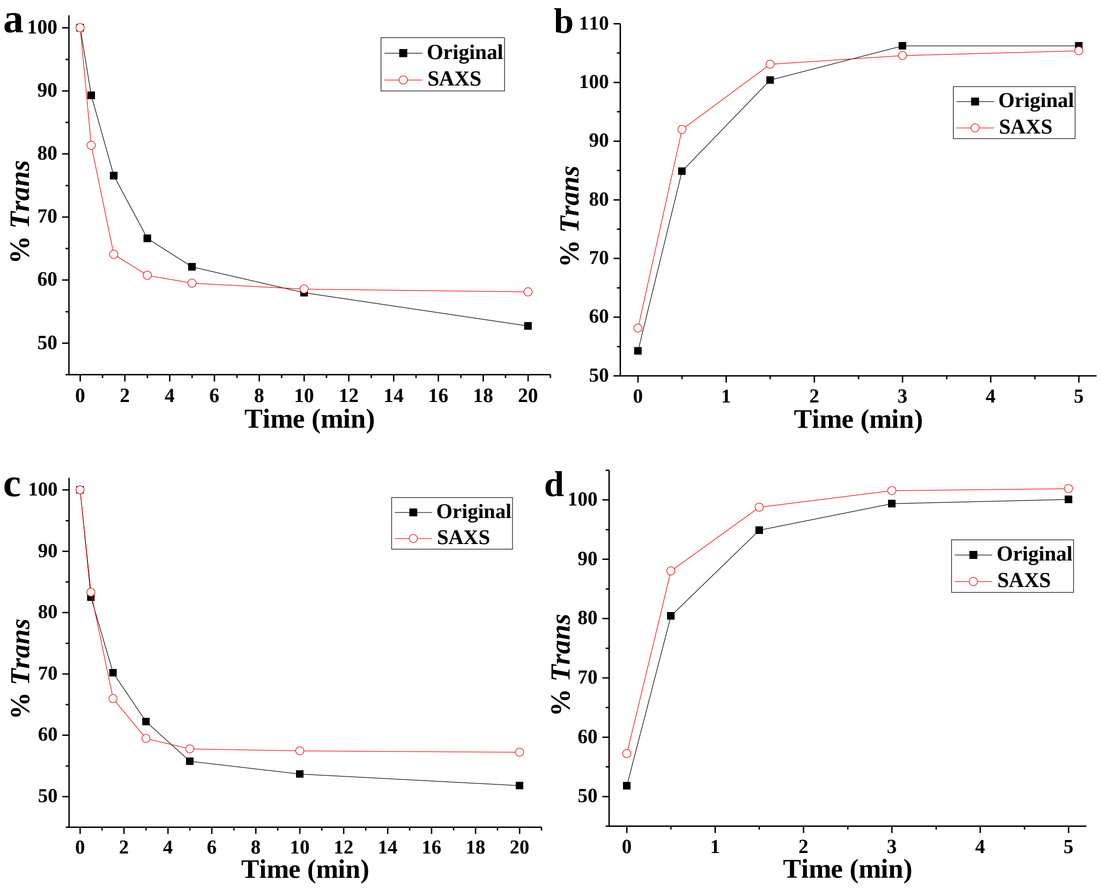

Figure S5. Photoisomerization of CAB15 (a, b) and CAB30 (c, d) before and after SAXS measurements. Percentage of *trans*-sample as a function of irradiation time for *trans*-to-*cis* (a, c) and *cis*-to-*trans* (b, d) isomerization.

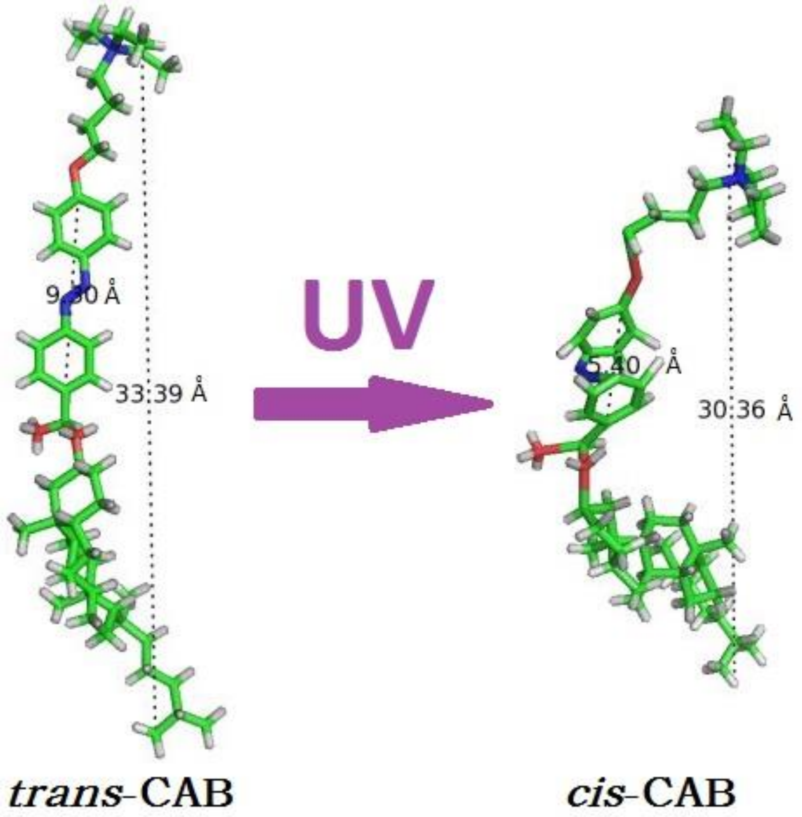

Figure S6. Molecular structures of *trans*/*cis*-CAB simulated by Pymol.

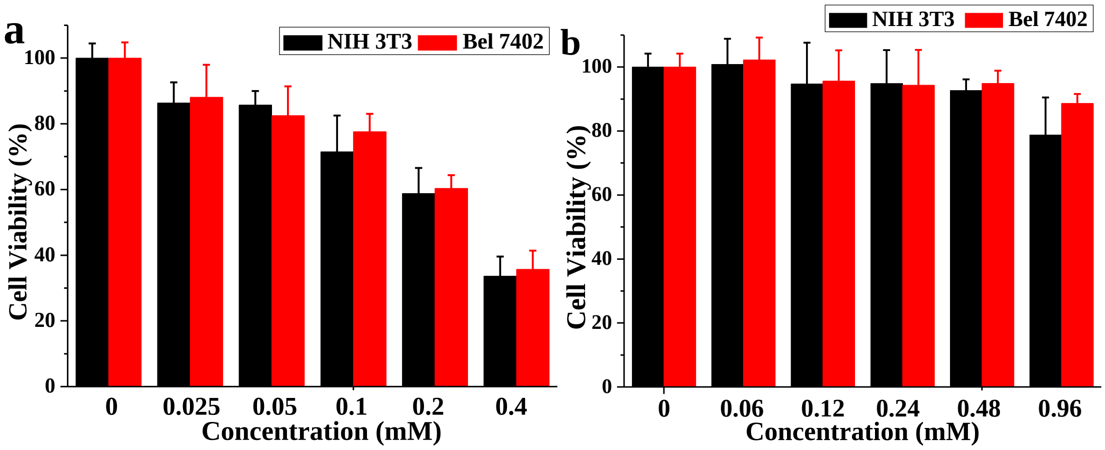

Figure S7. Cytotoxicity of individual SDS (a) or CAB (b) at 24 h treatment with NIH 3T3 cells and Bel 7402 cells. Cell viability was assessed by the MTT assay (n = 6).

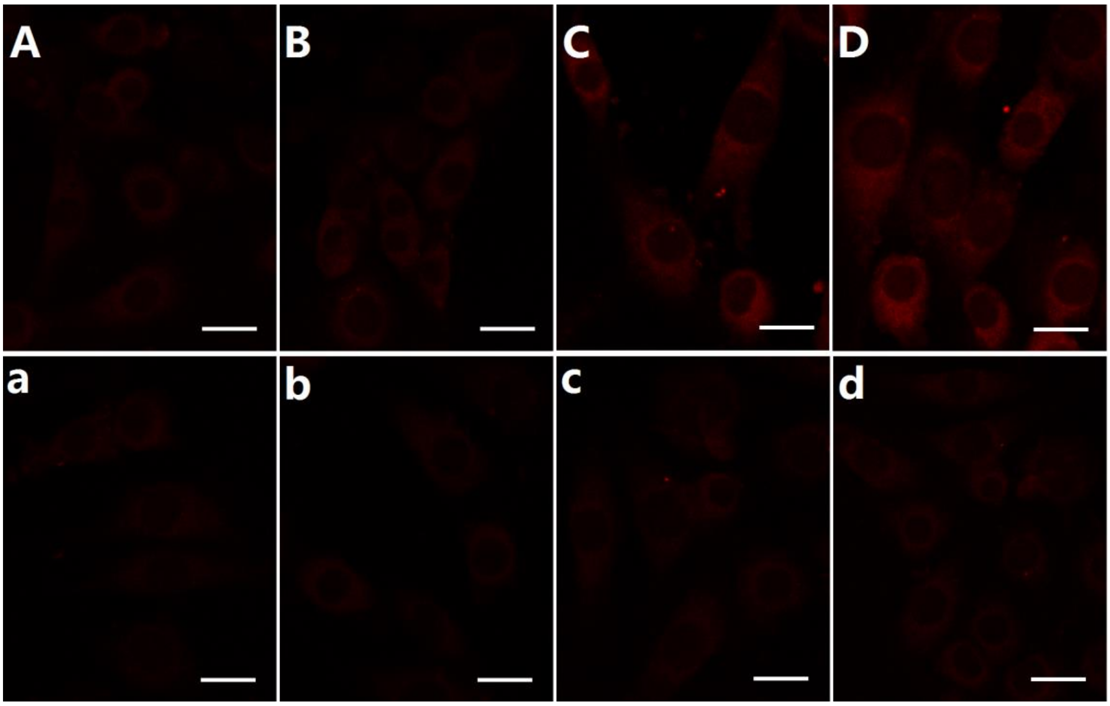

Figure S8. Confocal microscopy snapshots of NIH 3T3 cells incubated for different times at (A-D) 37 ℃ or (a-d) 4 ℃ with CAB15-RhB. (A, a) 5 min, (B, b) 15 min, (C, c) 30 min, and (D, d) 60 min. Fluorescence images were taken by Leica SP8. Scale bars are 20 μm.

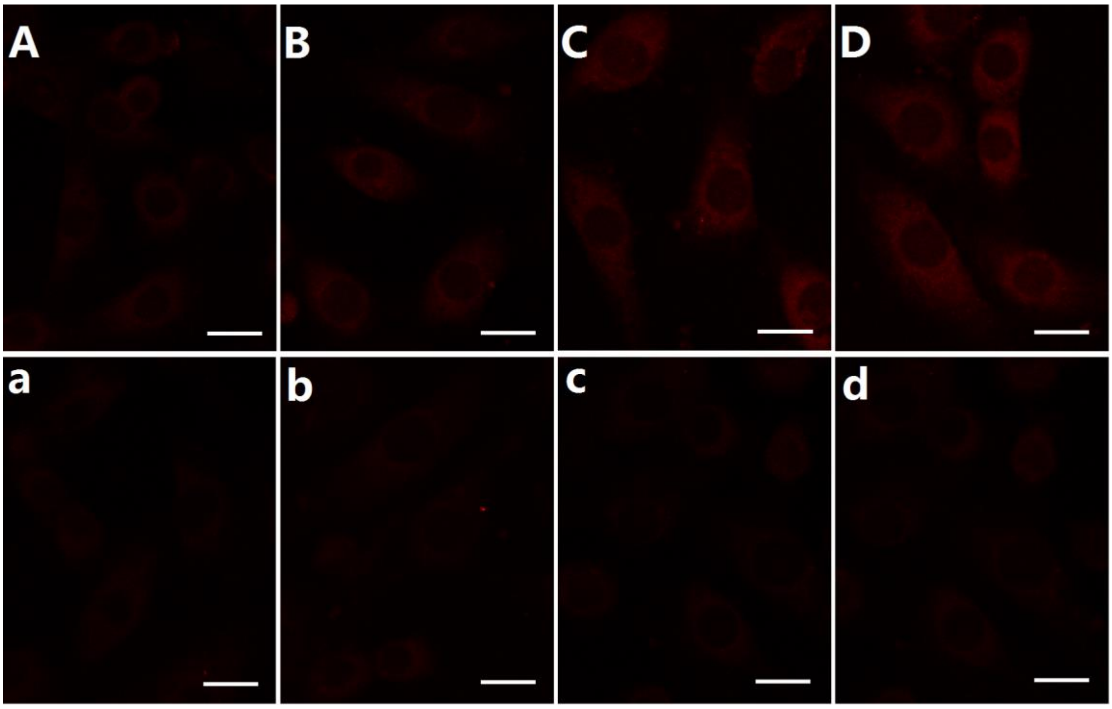

Figure S9. Confocal microscopy snapshots of NIH 3T3 cells incubated for different times at (A-D) 37 ℃ or (a-d) 4 ℃ with CAB30-RhB. (A, a) 5 min, (B, b) 15 min, (C, c) 30 min, and (D, d) 60 min. Fluorescence images were taken by Leica SP8. Scale bars are 20 μm.
